# *Capparis spinosa* reduces Doxorubicin-induced cardio-toxicity in cardiomyoblast cells

**Published:** 2016

**Authors:** Seyed Hadi Mousavi, Azar Hosseini, Elham Bakhtiari, Hassan Rakhshandeh

**Affiliations:** 1 *Pharmacological Research Center of Medicinal Plants, School of Medicine, Mashhad University of Medical Sciences, Mashhad, Iran*; 2 *Department of Pharmacology, School of Medicine, Mashhad University of Medical Sciences, Mashhad, Iran*; 3 *Medical Toxicology Research Center, School of Medicine, Mashhad University of Medical Sciences, Mashhad, Iran*

**Keywords:** *C. spinose*, *H9c2 cells*, *Doxorubicin*, *Apoptosis*

## Abstract

**Objective:**

Doxorubicin (DOX) is an effective anticancer drug but its clinical application is limited because it induces apoptosis in cardiomyocytes and leads to permanent degenerative cardiomyopathy and heart failure possibly due to oxidative stress. Recent studies showed that *Capparis spinosa *(*C. spinose*) exhibits potent antioxidant activity. So, in this study, we explored the protective effect of hydro-alcoholic extract of *C. spinosa *against DOX-induced cytotoxicity in H9c2 cells.

**Materials and Methods:**

Cell viability was quantified by MTT assay. Apoptotic cells were determined using flow cytometry (sub-G1 peak) evaluation of DNA fragmentation following PI staining. Cells were cultured with 5 μM DOX for 24 hr to induce cell damage. H9c2 cells were pretreated with different concentrations (6-200 μg/ml) of *C. spinosa *extract for 4 hr before DOX treatment in all trials.

**Results:**

Pretreatment with 25, 50, 100 and 200 µg/ml of *C. spinosa* could increase the viability of H9C2 cells to 72.63±2.8% (p<0.05), 77.37±1.8% (p<0.05), 83.56±2.6% (p<0.001) and 90.9±0.5% (p<0.001) of control, respectively. Also, *C. spinosa* decreased apoptotic induction significantly, at the doses of 50 µg/ml (p<0.05), 100 µg/ml (p<0.01) and 200 µg/ml (p<0.001)

**Conclusion:**

Our results showed that *C. spinosa *could exert cardioprotective effects against DOX-induced toxicity that might be mediated via its antioxidant activity.

## Introduction

Doxorubicin (DOX) is an anti-neoplastic drug. It is used for the treatment of variety of malignancies, such as leukemias, Hodgkin and non-Hodgkin lymphoma, and solid tumors (Wu et al., 2002 ▶). However, it leads to cardio-toxicity, so its clinical usage is limited. The accurate mechanisms of DOX-induced cardiotoxicity are not completely understood, but different studies indicate the generation of reactive oxygen species (ROS) involvement (Bryant et al., 2007 ▶). Interestingly, some natural foods have been reported to contain substantial amounts of antioxidants and free radical scavenging agents. These compounds diminish some side effects of chemotherapeutic agents on normal cells by reducing their genotoxicity (Bryant et al., 2007 ▶)*. Capparis spinosa *L. (*C. spinose*), the caper bush, is a perennial winter-deciduous plant. It is used as an anti-oxidative (Tlili et al., 2010 ▶; Siracusa et al., 2011 ▶), anti-inflammatory (Issac et al., 2011 ▶), anti-bacterial (Boga et al., 2011 ▶), anti-diabetic (Huseini et al., 2013 ▶), anti-hepatotoxic (Aghel et al., 2007 ▶), and anti-proliferative agent (Wu et al., 2003 ▶). Moreover, n-butanol extract of *C. spinosa* inhibits the growth of tumor cells (Yu-Bin and Lie 2014 ▶). It has anti-hyperglycemic and anti-obesity effects (Lemhadri et al., 2007 ▶). Also, its aqueous extract reduced cholesterol, triglycerides and glucose in normal and severe hyperglycemic rats (Eddouks et al., 2004 ▶, 2005). Clinical studies have shown that the caper extract has anti-arthritic effect (Panicoet al., 2005 ▶; Feng et al., 2011 ▶). Phytochemical studies have reported that the extract of *C. spinosa *contains antioxidant compounds such as flavonoids, quercetin and kaempferol glycosides (Argentieri et al., 2012 ▶). In this research, the protective effect of *C. spinosa* hydro-alcoholic extract was evaluated against DOX in cardiomyoblast cell line for the first time.

## Materials and Methods

**Reagents**

3-(4,5-dimethylthiazol-2-yl)-2,5-diphenyl tetrazolium (MTT), Propidium iodide (PI), sodium citrate and Triton X-100 were purchased from Sigma (St Louis, MO, USA). High-glucose Dulbecco’s Modified Eagles Medium (DMEM), penicillin-streptomycin and fetal bovine serum were purchased from Gibco. H9C2 cells (cardiomyoblast cells of rat) were obtained from Pasteur Institute (Tehran, Iran). DOX was purchased from EBEWE Company (Austria).

**Preparation of extracts**

The aerial parts of *C. spinosa *were collected from the Garden of Ferdowsi University, Mashhad, Iran. The plant sample was identified at the herbarium of school of Pharmacy (Mashhad University of Medical Sciences, Mashhad, Iran) and a specimen voucher (13063) was deposited. The aerial parts of *C. spinosa *were dried, powdered and 50 g of powder were subjected to extraction with 70% ethanol in a Soxhlet apparatus for 48 hr. The hydro-alcoholic extract was then dried on a water bath and the remaining (yield percentage 20% w/w) was dissolved in DMSO and kept frozen at -18 ^o^C (Hosseini et al., 2014 ▶).

**Cell culture**

H9c2 Cells were maintained at 37^o^C in a humidified atmosphere containing 5% CO_2_. The cells were cultured in DMEM supplemented with 10% fetal bovine serum and 100Units/ml penicillin and 100 µg/ml streptomycin. For MTT experiments, cells were seeded in 96-well culture plates. For the apoptosis assay, cells were seeded at 1×10^5^/well in a 24-well plate. All treatments were carried out in triplicate. Cells were pretreated with the extract (6-200 µg/ml) for 4 hr and then incubated with the extract and 5 µM doxorubicin for 24 hr.

**Cell viability**

The cell viability was determined using a modified MTT assay as described previously (Hosseini et al., 2014 ▶). Briefly, MTT solution in phosphate-buffered saline (5 mg/ml) was added to each well at a final concentration of 0.05%. After 3 hr, the formazan precipitate was dissolved in DMSO. The absorbance at 570 and 620 nm (background) was measured using a StatFAX303 plate reader. 

**Apoptosis**

Apoptotic cells were detected using PI staining of small DNA fragments followed by flow cytometry. It has been reported that a sub-G1 peak that is reflective of DNA fragmentation can be observed following the incubation of cells with a hypotonic phosphate-citrate buffer containing a quantitative DNA-binding dye, such as PI. Apoptotic cells that have lost DNA will take up less stain and appear on the left side of the G1 peak in the histogram (Bakhtiari et al., 2015 ▶). Briefly, after treatment of the cells with agents floating and adherent cells were then harvested and incubated at 4 °C overnight in the dark with 750 µl of a hypotonic buffer (50 µg/ml PI in 0.1% sodium citrate with 0.1% Triton X-100). Next, flow cytometry was carried out using a FACScan flow cytometer (Becton Dickinson). A total of 1×10^4^ events were acquired with fluorescence-activated cell sorting (FACS).

**Results**

**Effect of** ***C. spinosa*** **extract on cell viability**

**Figure 1:**
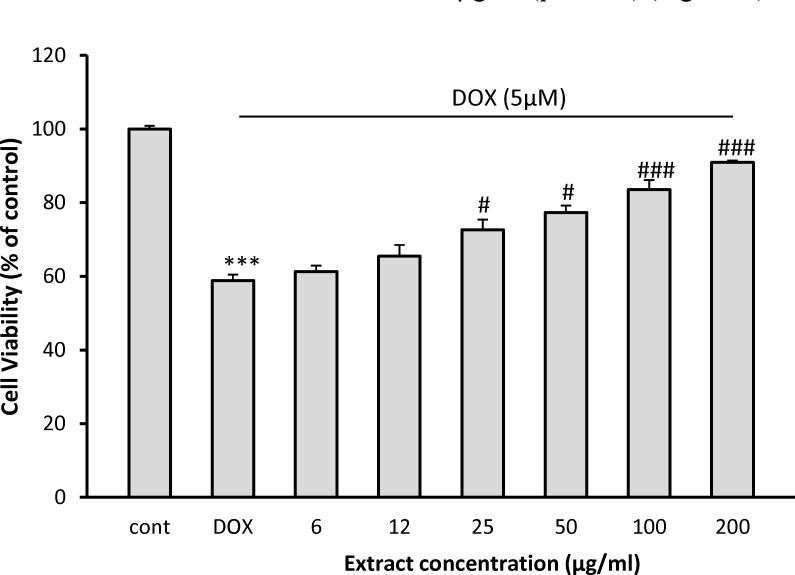
Effect of *C. spinosa* extract on H9c2 cells viability exposed to DOX for 24 hr. The percentage cell viability (quantitated by MTT assay) was normalized against the control. ^***^p< 0.001 versus control, ^###^p<0.001, ^#^p<0.05 versus DOX

Incubation with DOX significantly decreased cell viability to 58.8 ± 1.6% of control (p<0.001). Pretreatment with 25, 50, 100 and 200 µg/ml of *C. spinosa* could increase the viability of H9C2 cells to 72.63 ± 2.8% (p< 0.05), 77.37 ± 1.8% (p< 0.05), 83.56 ± 2.6% (p< 0.001) and 90.9 ± 0.5% (p< 0.001) of control, respectively (Figure 1). At the doses of 6 and 12 µg/ml, however, *C. spinosa* was not able to protect H9C2 cells against DOX-induced cytotoxicity.

**Effect of** ***C. spinosa*** **on apoptotic induction**

Apoptosis in H9C2 cell line was detected by flow cytometry using PI staining (Figure2a). Analysis of the sub-G1 peak in flow cytometry histograms revealed the induction of apoptosis in cells treated with DOX (p<0.001). *C. spinosa* decreased apoptotic induction significantly, at the doses of 50 µg/ml (p<0.05), 100 µg/ml (p<0.01) and 200 µg/ml (p<0.001) (Figure2b). 

**Figure 2a:**
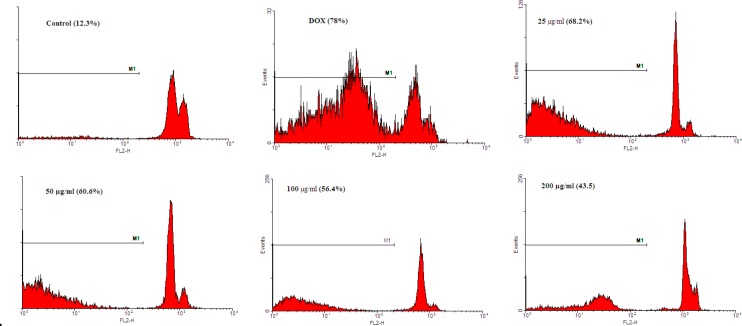
The effects of the *C. spinosa* extract on apoptosis in H9c2 cells using PI staining and flow cytometry

**Figure 2b:**
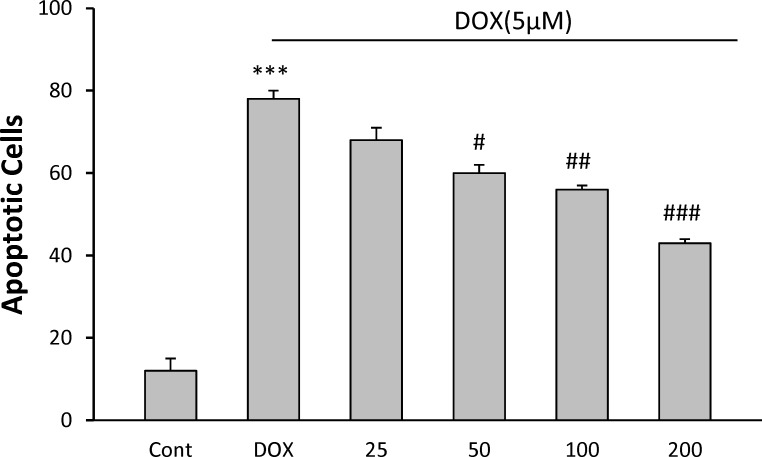
The effects of the *C. spinosa* extract on apoptosis in H9c2 cells using PI staining and flow cytometry. ^***^p< 0.001 versus control, ^#^p<0.05, ^##^p<0.001, ^###^p<0.001, versus DOX.

## Discussion

Despite the development of several anti-tumor drugs, DOX is widely used as a chemotherapeutic agent. In recent years, its clinical usage is limited because of its serious dose-dependent cardiotoxicity. DOX induces cardio-toxicity via generation of free radicals and depletion of endogenous antioxidants (Takemura and Fujimara, 2007 ▶). Oxidative stress occurs when the production of ROS is more than the capacity of antioxidant defense systems such as glutathione peroxidase, catalase and superoxide dismutase (SOD) (Li et al., 2013 ▶). The low antioxidant enzyme activity in cardiomyocytes may be a cause for their sensitivity to oxidative injury (Kang et al., 1996 ▶). So, employing pharmacological approaches to decrease oxidative stress in the heart is a favorable method for cardioprotection (Li et al., 2013 ▶). The results indicated that *C. spinosa* has protective effect in H9c2 cells against DOX-induced oxidative stress. H9c2 cells are morphologically alike immature embryonic cardiomyocytes but they are functionaly similar to adult cardiac cells (Sheng et al., 2010 ▶). However, they are a suitable model for studying oxidative stress-induced cardiomyocyte injury (Winstead et al., 2005 ▶). For the first time, the protective effect of *C. spinosa* against DOX-induced cell death was studied in H9c2 cells. In this research, pretreatment with* C. spinosa* protected cells in a concentration-dependent manner. *C. spinosa* could increase cell viability and decrease cell apoptosis. These effects may be partly attributed to antioxidant activity.

Phytochemical studies have reported that different parts of this herb contain antioxidant compounds such as phenols and flavonoids (Tesriere et al., 2007 ▶). The antioxidant potential of *C. spinosa* can scavenge free radicals and reduce oxidative stress (Rashedi et al., 2015 ▶). *In vitro* studies have revealed antioxidant activity of *C. spinosa*. The total alkaloids of *C. spinosa* can inhibit the growth of human gastric adenoma cells SGC-7901 (Yu et al., 2008 ▶). Aqueous and methanolic extract of *C. spinosa* root inhibited the growth of AMN3 cells (Al-Asady, 2007 ▶). The lectin isolated from seeds of *C. spinosa* inhibited the proliferation of both HepG2 and MCF-7 cell lines (Lam et al., 2009 ▶). Recent studies have revealed *C. spinosa* induced apoptosis via mitochondrial cytochrome c release and caspase-9 and caspase-3 activation in cancer cell lines (Yu-Bin and Lei, 2014 ▶).

## Note

*Please cite this paper as: *

Mousavi SH, Hosseini A, Bakhtiari E, Rakhshandeh H. *Capparis spinosa* reduces Doxorubicin-induced cardio-toxicity in Cardiomyoblast cells.Avicenna J Phytomed, 2016; 6 (5): 489-494.
